# Cholesteryl ester transfer protein inhibitors: from high-density lipoprotein cholesterol to low-density lipoprotein cholesterol lowering agents?

**DOI:** 10.1093/cvr/cvab350

**Published:** 2021-11-26

**Authors:** Nick S Nurmohamed, Marc Ditmarsch, John J P Kastelein

**Affiliations:** Department of Vascular Medicine, Amsterdam UMC, University of Amsterdam, Meibergdreef 9, 1105 AZ, Amsterdam, The Netherlands; Department of Cardiology, Amsterdam UMC, Vrije Universiteit Amsterdam, Boelelaan 1117, 1081 HV, Amsterdam, TheNetherlands; NewAmsterdam Pharma, Gooimeer 2-35, 1411 DC Naarden, The Netherlands; Department of Vascular Medicine, Amsterdam UMC, University of Amsterdam, Meibergdreef 9, 1105 AZ, Amsterdam, The Netherlands

**Keywords:** CETP, CETP inhibitor, ASCVD, LDL-C, HDL-C

## Abstract

Cholesteryl ester transfer protein (CETP) is a liver-synthesized glycoprotein whose main functions are facilitating transfer of both cholesteryl esters from high-density lipoprotein (HDL) particles to apolipoprotein B (apoB)-containing particles as well as transfer of triglycerides from apoB-containing particles to HDL particles. Novel crystallographic data have shown that CETP exchanges lipids in the circulation by a dual molecular mechanism. Recently, it has been suggested that the atherosclerotic cardiovascular disease (ASCVD) benefit from CETP inhibition is the consequence of the achieved low-density lipoprotein cholesterol (LDL-C) and apoB reduction, rather than through the HDL cholesterol (HDL-C) increase. The use of CETP inhibitors is supported by genetic evidence from Mendelian randomization studies, showing that LDL-C lowering by *CETP* gene variants achieves equal ASCVD risk reduction as LDL-C lowering through gene proxies for statins, ezetimibe, and proprotein convertase subtilisin–kexin Type 9 inhibitors. Although first-generation CETP inhibitors (torcetrapib, dalcetrapib) were mainly raising HDL-C or had off-target effects, next generation CETP inhibitors (anacetrapib, evacetrapib) were also effective in reducing LDL-C and apoB and have been proven safe. Anacetrapib was the first CETP inhibitor to be proven effective in reducing ASCVD risk. In addition, CETP inhibitors have been shown to lower the risk of new-onset diabetes, improve glucose tolerance, and insulin sensitivity. The newest-generation CETP inhibitor obicetrapib, specifically designed to lower LDL-C and apoB, has achieved significant reductions of LDL-C up to 45%. Obicetrapib, about to enter phase III development, could become the first CETP inhibitor as add-on therapy for patients not reaching their guideline LDL-C targets.

## 1. Introduction

The number of low-density lipoprotein (LDL) and other apolipoprotein B (apoB)-containing particles has firmly been established as causal risk factor for atherosclerotic cardiovascular disease (ASCVD). In the last half century, a massive body of evidence from studies in animals and humans has accumulated to support this conclusion. Large observational studies, randomized clinical trials, as well as more recent Mendelian randomization studies in hundreds of thousands of participants have confirmed LDL cholesterol (LDL-C) and apoB as one of the most important risk factors for ASCVD.

In addition, there is robust evidence that reducing the plasma concentration of LDL-C reduces the risk of ASCVD.^1^ In line with the causal relation between apoB and ASCVD, it has been shown that the ASCVD risk reduction of lipid-lowering therapies is proportional to the magnitude and duration of the reduction of apoB-containing particles. Over the years, the cornerstone of lipid-lowering therapies has been laid down in the form of statin therapy, ezetimibe, and proprotein convertase subtilisin–kexin Type 9 (PCSK9) inhibitors, and most recently, bempedoic acid has broadened the therapeutic options.^2^ However, there remains a significant lipid-driven residual burden of ASCVD, particularly following data-driven implementation of more stringent LDL-C guideline goals, especially for patients at high- and very high risk for future cardiovascular events. In fact, a recently conducted EU-wide cross-sectional observational study revealed that overall risk-based 2019 LDL-C goal attainment to less than 1.4 mmol/L was observed in just 18% of very high-risk patients.^3^ In particular, in very high-risk patients receiving statin monotherapy, goal attainment was 14%, 16%, and 22% in those receiving low-, moderate-, and high-intensity statins, respectively. This is largely due to underuse, low adherence, and intolerance issues associated with statins and ezetimibe.^4,5^ The introduction of PCSK9 inhibitors has been limited by reimbursement restrictions till today as well as by the burden of injections, whereas bempedoic acid is a modest LDL-C-lowering option. Therefore, there is still a need for additional and convenient lipid-lowering therapies.

The first three cholesteryl ester transfer protein (CETP) inhibitors (torcetrapib, dalcetrapib, and evacetrapib) have not shown a reduction of ASCVD events in multiple large Phase 3 clinical trials. However, these trials were hampered by either off-target effects seen with torcetrapib, lack of LDL-C lowering with dalcetrapib or limited follow-up with evacetrapib.^6^ Conversely, genotypes at the *CETP* locus that are associated with low CETP activity have shown an equal ASCVD risk reduction per unit of apoB-lowering compared to *HMGCR, PCSK9*, and *NPC1L1* genotypes using Mendelian randomization. In addition, the Randomized EValuation of the Effects of Anacetrapib through Lipid-modification (REVEAL) investigators have recently presented their long-term follow-up trial data, revealing a 20% major cardiovascular event (MACE) reduction in the 6.4 year following period.^7^ It remains to be established whether a more potent CETP inhibitor specifically designed to lower LDL-C and apoB-containing lipoproteins could give rebirth to the CETP inhibitor class. This review focuses on the functions of CETP, the role of CETP in atherosclerosis, the different CETP inhibiting agents developed, and the future of CETP inhibition.

## 2. Functions of CETP

**Figure 1:**
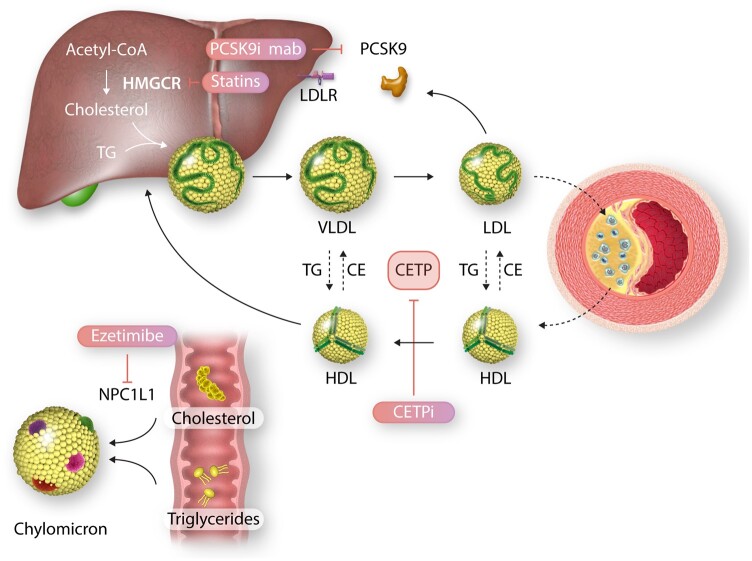
Overview of working mechanisms of CETP inhibition and traditional lipid-lowering therapies. Simplified overview of cholesterol metabolism. Cholesteryl ester transfer protein (CETP) facilitates transfer of cholesteryl esters (CE) and triglycerides (TG) between lipoproteins. Transfer of CE to VLDL particles contributes to maturation to LDL particles, which contribute to foam cell formation in the atherosclerotic plaque. Cholesteryl ester transfer protein inhibitors (CETPi) impair transfer of cholesterol esters from high-density lipoprotein (HDL) to apoB particles and transfer of triglycerides from apoB to HDL particles. Proprotein convertase subtilisin–kexin Type 9 inhibiting (PCSK9i) monoclonal antibodies block PCSK9 binding to low-density lipoprotein receptor (LDLR). Statins block 3-hydroxy-3-methylglutaryl coenzyme reductase (HMGCR). Ezetimibe inhibits Niemann-Pick-like protein 1C1 (NPC1L1), preventing transport of sterols into enterocytes.

CETP is a glycoprotein that is synthesized in the liver and promotes bidirectional transfer of cholesteryl esters and triglycerides between all plasma lipoprotein particles: (i) transfer of cholesteryl esters from cholesteryl ester-rich high-density lipoprotein (HDL) particles to LDL and very LDL (VLDL) particles and (ii) transfer of triglycerides from triglyceride-rich VLDL particles and chylomicrons to HDL and LDL particles (*Figure 1*).^8–11^ Thereby, CETP has a direct effect on both plasma HDL cholesterol (HDL-C) as well as LDL-C levels. CETP has a boomerang shape, has cavities at either end that enable binding of both cholesteryl esters and triglycerides and a tunnel spanning the entire length of the molecule.^12^ The majority of the cholesteryl esters found in plasma are formed within HDL particles, while VLDL particles and chylomicrons are the major carrier of triglycerides.

**Figure 2:**
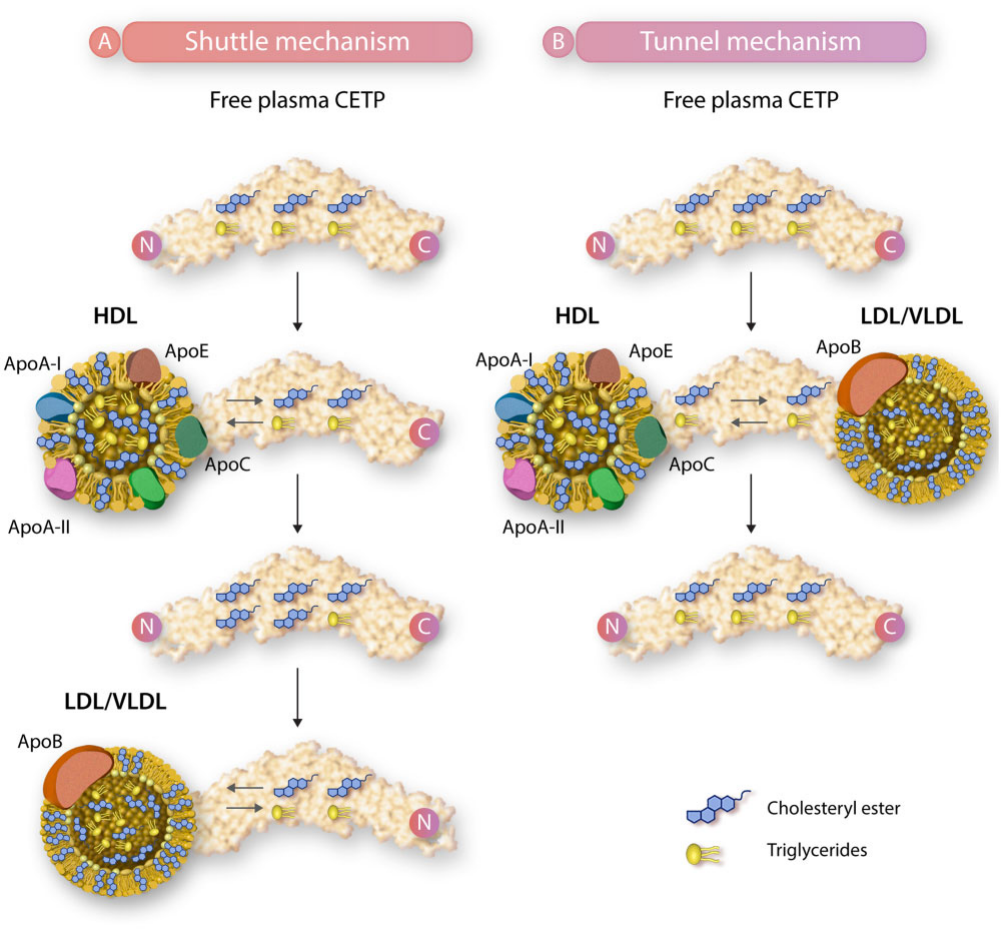
Mechanisms of cholesteryl esters and triglycerides transfer by CETP. CETP facilitates bidirectional transfer of cholesteryl esters and triglycerides via two known mechanisms. The first (*A*) is a shuttle mechanism where CETP binds a lipoprotein (shown is HDL) particle, exchanging cholesteryl esters and triglycerides (for HDL: cholesteryl esters out, triglycerides in). After detaching, the CETP molecule binds to a second lipoprotein particle (shown is LDL/VLDL), again exchanging cholesteryl esters and triglycerides (for LDL/VLDL: cholesteryl esters in, triglycerides out). The second (*B*) mechanism is a tunnel mechanism. The N-terminal domain binds to an HDL particle forming a CETP–HDL complex, which binds to either an LDL or VLDL particle through the C-terminal domain, forming a ternary complex between HDL, CETP, and LDL or VLDL. CETP, cholesteryl ester transfer protein; HDL, high-density lipoprotein; LDL, low-density lipoprotein; VLDL, very low-density lipoprotein.

There are two mechanisms by which CETP is thought to facilitate transfer of triglycerides and cholesteryl ester between the different plasma lipoprotein fractions in plasma (*Figure 2*). The first is a shuttle mechanism where CETP randomly binds a lipoprotein particle, forming a complex to exchange triglycerides and cholesteryl with the particular lipoprotein particle.^13^ Then, this CETP molecule detaches from the lipoprotein particle and freely circulates through the plasma until it finds a new lipoprotein particle (either in the same or in a different lipoprotein fraction) to bind to. CETP will then again exchange triglycerides and cholesteryl esters with the second lipoprotein particle forming another transient complex. In this way, CETP promotes an equilibrium of both cholesteryl esters and triglycerides between all plasma lipoprotein particles. The second mechanism by which CETP is thought to transfer cholesteryl esters and triglycerides is a tunnel mechanism. The N-terminal domain of CETP initially penetrates the HDL particle surface forming a CETP–HDL binary complex.^14,15^ Then, this complex will form a ternary complex (a complex between two substrate molecules and a protein) with either an LDL or VLDL particle through the C-terminal domain of CETP. So, in this way, a ternary complex between CETP, HDL, and an LDL or VLDL particle is formed. Molecular forces caused by both lipoproteins bound at either end of the CETP molecule result in twisting of the CETP molecule. Subsequently, this results in opening of a tunnel through which cholesteryl esters are transferred from HDL particles to either LDL or VLDL particles and triglyceride is transferred from a VLDL particle to an HDL particle. After this transfer, the ternary complex dissociates and CETP, the HDL particle, and the LDL or VLDL particle can circulate in plasma freely. As a result of this transfer, the VLDL or LDL particle is enriched in cholesteryl esters and depleted of triglycerides and the HDL particle is depleted of cholesteryl esters and enriched in triglycerides, a situation that generally associates with a pro-atherogenic state in humans. Available evidence suggests that both the first shuttle mechanism as well as the second tunnel mechanism operate simultaneously to redistribute cholesteryl esters and triglyceride between the different plasma lipoprotein fractions.

## 3. The role of CETP in atherosclerosis

By evolution, CETP is present and active in all primates, rabbits, and hamsters, but is lacking in the plasma of most other species.^16^ Importantly, CETP is absent in the plasma of multiple species that are frequently used for studying atherosclerosis such as rodents, dogs, and pigs. It is hypothesized that species which possess CETP, such as rabbits, are much more susceptible to the development of atherosclerosis than species that do not possess CETP. In the first part of the 20th century, most investigations into the role of cholesterol in the development of atherosclerosis were performed in rabbits on a high cholesterol, egg-yolk diet. Conversely, rodents, which lack CETP, are naturally resistant to the development of atherosclerosis. Introduction of the *CETP* gene into mice increased plasma LDL-C levels, while plasma HDL-C levels were decreased, predisposing the mice to the development of atherosclerosis.^17–19^ Transgenic expression of the *CETP* gene has proven to be pro-atherogenic in apolipoprotein (apo)E knock-out mice,^18^ in mice fed an atherogenic diet,^19^ in LDL receptor knock-out mice,^18^ in APOE*3-Leiden mice,^17^ and in hypertensive rats.^20^ In a study of APOE*3-Leiden expressing CETP, treatment with anacetrapib dose-dependently reduced atherosclerosis and improved lesion stability.^21^ These effects were mainly caused by a reduction in the plasma concentration of non-high-density lipoprotein cholesterol (non-HDL-C).

As mentioned earlier, rabbits, in contrast to rodents, have a high plasma CETP level and are susceptible to the development of atherosclerosis induced by diet.^16^ Multiple strategies to inhibit CETP in rabbits led to a reduction of the development of atherosclerosis. These strategies include administration of the small molecule CETP inhibitors, dalcetrapib^22^ and torcetrapib,^23^ a CETP antisense oligonucleotide,^24^ and an anti-CETP vaccine.^25^ However, the dalcetrapib data were later challenged by the group of Mabuchi in another cholesterol-fed rabbit model, which supports the contention that weak CETP inhibitors that only raise HDL-C have no effect on atherosclerosis.

## 4. Epidemiology and genetics of CETP and CETP inhibition

The observation in Japan that mutations in the *CETP* gene led to markedly increased HDL-C plasma levels as well as reduced LDL-C plasma levels in several families first sparked interest in pharmacological inhibition of CETP.^26,27^ Subsequently, CETP inhibitors were developed which primarily raised HDL-C plasma levels, whereas the more potent CETP inhibitors developed later also lowered LDL-C plasma levels. Before large-scale clinical outcome trials with these CETP inhibitors were performed, the hypothesis was that increases in HDL-C, which were epidemiologically strongly associated with ASCVD, would result in a potent reduction in MACE rates. However, merely one (REVEAL) out of four cardiovascular outcome trials (CVOTs) performed, demonstrated a significant reduction in MACE. Conversely, it was shown that this reduction was not the consequence of increases in HDL-C but was rather associated with the reductions in LDL-C or non-HDL-C.^28^

Human genetic studies have shown that CETP is pro-atherogenic and that genetically lower CETP activity is associated with a lower ASCVD risk. Both large meta-analyses^29,30^ and cohort studies^31,32^ have taught us that certain *CETP* gene polymorphisms are associated with decreased CETP activity. As a consequence, concordant effects on lipoprotein concentrations were associated with a reduced risk of having an ASCVD event. In the Copenhagen City Heart Study, which included 10 261 participants, two common *CETP* gene polymorphisms reducing CETP activity were associated with significant reductions in the risk of ischaemic heart disease, myocardial infarction (MI), ischaemic cerebrovascular disease, and ischaemic stroke.^31^ In addition, it was shown that the number of alleles with gene polymorphisms determined the impact on ASCVD risk. Furthermore, participants with these polymorphisms demonstrated longevity, whereas there was no evidence of adverse effects associated with mutations in the *CETP* gene. In contrast, insights from Mendelian randomization of SNPs in other genes associated with isolated changes in HDL-C have shown no association with ASCVD.^33^ Therefore, it is hypothesized that reductions in ASCVD risk associated with polymorphisms in the *CETP* gene are achieved by a reduction of LDL-C and other atherogenic lipoproteins rather than an increase in HDL-C levels. This belief was confirmed in a study of CETP truncating mutations, in which the magnitude of the benefit on ASCVD was strongly correlated with the degree of LDL-C lowering.^6^

**Figure 3:**
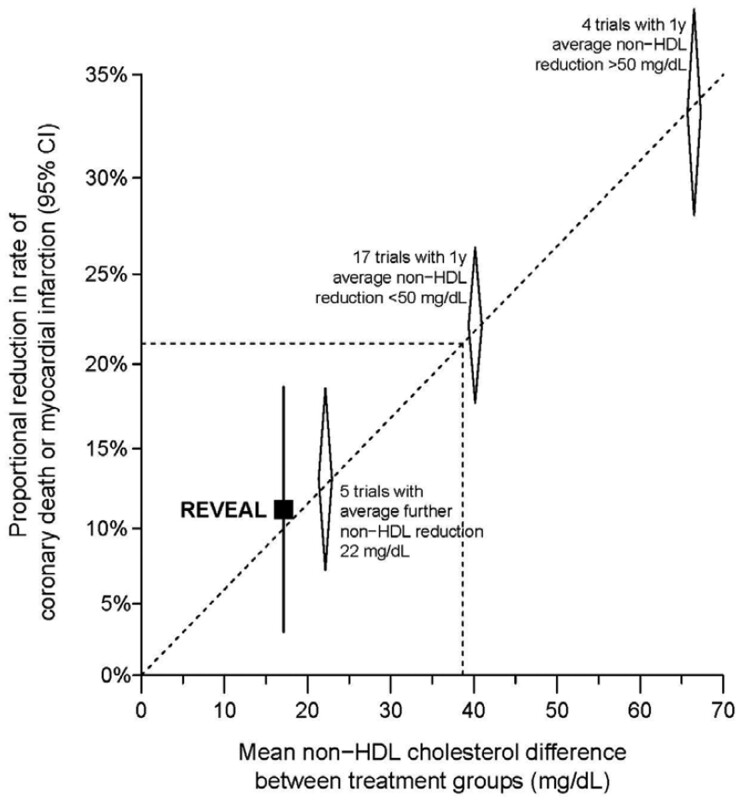
REVEAL trial and statins in the CTT meta-analysis. Reduction in rate of coronary death or myocardial infarction from the REVEAL trial, compared reduction in statin trials from the CTT, plotted according to the size of the absolute reduction in non-HDL cholesterol. Adapted from Bowman *et al.*,^58^ Copyright © 2021 Massachusetts Medical Society. Reprinted with permission from REVEAL, Randomized EValuation of the Effects of Anacetrapib through Lipid-modification. CTT, Cholesterol Treatment Trialists’; HDL, high-density lipoprotein.

These findings were further confirmed in a large Mendelian randomization analysis by Ference *et al*.^34^ including 102 837 participants from cohort and case–control studies from North America and the UK. In this analysis, Ference *et al.*^34^ investigated the association between *CETP* scores, changes in lipid and lipoprotein levels, and the effect on ASCVD event rate was further validated in an additional 189 539 participants. It was indeed shown that variants in the *CETP* gene were associated with higher HDL-C levels and concordant reductions of LDL-C and apoB, which were associated with a lower risk of ASCVD events. Interestingly, per unit change in LDL-C and apoB levels, this risk reduction was of the same magnitude as seen with other genetic variants associated with targets of LDL-lowering therapies (*HMGCR, NPC1L1*, and *PCSK9*). Extrapolated to pharmacological inhibition of CETP, it can be expected that the ASCVD benefit per mmol/L lowering of LDL-C achieved with CETP inhibitors is equal to the ASCVD benefit of other lipid-lowering therapies such as statins, ezetimibe, and PCSK9 inhibitors. In line with these Mendelian randomization studies, results from the REVEAL trial evaluating anacetrapib also showed that the ASCVD benefit from CETP inhibition might be the consequence of achieved LDL-C and apoB reduction and is proportional to the benefit of other lipid-lowering therapies (*Figure 3*).^35^ This means that for every mmol/L (38.67 mg/dL) of LDL-C reduction, it could be inferred that ASCVD risk is reduced with approximately 22%.^1^ Collectively, during the last decade, these data combined underlie the important contention that the main ASCVD benefit from CETP inhibition is associated with the achieved LDL-C and apoB reduction, rather than with the achieved HDL-C increase.

## 5. Properties and effects of agents in the CETP inhibitor class

### 5.1 Molecular characteristics

As described previously, the main function of CETP is the transfer of cholesteryl esters and triglycerides between plasma lipoprotein particles. CETP inhibitors target a part of this mechanism by blocking transfer of cholesteryl esters. These inhibitors bind deeply within CETP, shifting the bound cholesteryl ester in the N-terminal pocket of the long hydrophobic tunnel and displacing the phospholipid from that pocket. At the opposite site, in the C-terminal pocket of the hydrophobic tunnel, the lipids remain unchanged. CETP inhibitors are positioned towards the narrow neck of the hydrophobic tunnel, and thereby inhibit the connection between C- and N-terminal pockets.

CETP inhibitors, which are highly lipophilic, bind mainly through extensive hydrophobic interactions with the protein and the shifted cholesteryl ester molecule. Enhanced understanding of the binding site has provided opportunities to design novel CETP inhibitors. It has been postulated that development of novel CETP inhibitors possessing more drug-like physical properties, i.e. less lipophilic, and could increase the aqueous solubility in plasma, which is very low with classical CETP inhibitors requiring non-traditional formulations for good oral absorption.^36^ To this end, obicetrapib was developed as a tetrahydroquinoline derivative possessing a pyrimidine and an ethoxycarbonyl structure with two chiral centres. Obicetrapib exhibits a cLogD_ph7.4_ of 4.9, whereas all other CETP inhibitors have a cLogD_ph7.4_ above 7 (for example anacetrapib: cLogD_ph7.4_ of 9.2).

### 5.2 Adipose tissue accumulation

Anacetrapib, the most lipophilic CETP inhibitor, has an unusually long elimination half-life. The early-phase clinical studies demonstrated a terminal elimination relatively short half-life up to 80 h after 14 days of dosing. However, subsequent studies demonstrated a much longer elimination half-life as treatment duration increased. After 8 weeks of treatment, termination half-life increased to 3–4 weeks, whereas after 76 weeks of dosing in the DEFINE study, anacetrapib remained detectable in plasma 2–4 years after the last dose.^37^ Moreover, anacetrapib was shown to accumulate in adipose tissue in much higher concentrations than in plasma in studies with subcutaneous adipose biopsy measurements.^38,39^ The same was observed with other very lipophilic drugs, such as amiodarone. While plasma levels of anacetrapib reached a plateau phase after 4 weeks of treatment, adipose tissue accumulation increases from 14-fold above plasma levels at 4 weeks to 64-fold above plasma by 16 weeks of dosing. Accumulation of anacetrapib in adipose tissue was not only more prolonged in plasma, but the accumulation in adipose tissue was also much more rapid than its elimination from adipose tissue. Thus, a combination of prolonged accumulation into and slow redistribution out of adipose tissue appears to cause the observed long terminal half-life of anacetrapib.

Interestingly, the other CETP inhibitors, which are also rather lipophilic, do not accumulate in adipose tissue. For example, the terminal half-life for torcetrapib is 211 h.^40^ For dalcetrapib, the terminal half-life is 30 h,^41^ whereas the terminal half-life for evacetrapib is approximately 40 h in studies of healthy subjects.^42^ For obicetrapib, the terminal half-life ranges between 121 and 151 h over the doses of 1–25 mg, also in healthy subjects.^43^ In the TULIP study, where patients were dosed for 12 weeks with obicetrapib 1, 2.5, 5, and 10 mg, pharmacokinetic sampling was performed up to 8 weeks post-dosing, it was shown that obicetrapib plasma concentrations had decreased by approximately 97% in all treatment groups at 8 weeks post-dosing.^44^

### 5.3 Effect on HDL-C, LDL-C, and apoB levels

The primary effect of CETP inhibition is a reduction of the rate of transfer of cholesteryl ester from HDL into triglyceride-rich lipoproteins, increasing HDL-C levels. Depletion of cholesteryl esters in the triglyceride-rich lipoproteins including VLDL, LDL, chylomicrons, and their remnants also leads to a decrease in VLDL and LDL apoB levels.^6^ CETP inhibitors were primarily developed to increase HDL-C levels, whereas the mechanism by which CETP inhibition reduces LDL-C was, initially, poorly understood. A study of apoB kinetics after 120 mg of torcetrapib, with or without atorvastatin, which was given to subjects with dyslipidaemia, demonstrated that torcetrapib reduced LDL apoB by increasing its fractional catabolic rate (FCR). In addition, Millar *et al.*^45^ conducted a larger study in which mildly hypercholesterolemic subjects received 100 mg anacetrapib added to their background treatment of statin or placebo for 8 weeks. It was shown that anacetrapib reduced LDL-C levels by increasing the LDL-apoB FCR, both in patients with placebo and statin background treatment. This points to a common mechanism driving enhanced LDL-apoB clearance, which reduces the total number of LDL particles and contributes to the reductions in LDL-C and apoB levels. Next, it was shown that anacetrapib reduces the total cholesterol content of LDL particles, which may contribute to the overall reduction in LDL-C levels.^45^ Collectively, these studies provided major contributions to understanding the mechanism by which CETP inhibitors reduce LDL-C levels.

The magnitude of LDL-C level lowering and effect on HDL-C levels of the different CETP inhibitors is shown in *Table 1*.

**Table 1:** Per cent changes from baseline for LDL-C and HDL-C as conferred by CETP inhibitors

CETP inhibitor	Dose (mg)	LDL-C (mmol/L) % change from baseline	HDL-C (mmol/L) % change from baseline	Years	References
Torcetrapib	60	–15.7	33.1	2006	^71^
Dalcetrapib	600	–5.4	26.4	2009	^86^
Anacetrapib	100	–23.4	138.1	2010	^1,79^
Evacetrapib	100	–22.3	94.6	2011	^87^
Obicetrapib	5	–45.3	157.1	2015	^44^

Shown is the change in LDL-C and HDL-C levels of the different CETP inhibitors.

CETP, cholesteryl ester transfer protein; HDL-C, high-density lipoprotein cholesterol; LDL-C, low-density lipoprotein cholesterol.

### 5.4 Effect on risk of new-onset diabetes

With the publication of Justification for the Use of Statin in Primary Prevention: An Intervention Trial Evaluating Rosuvastatin (JUPITER),^46^ the relationship between statin therapy and the development of Type 2 diabetes was first established. JUPITER was a CVOT investigating rosuvastatin 20 mg vs. placebo in more than 17 000 participants during a median follow-up of 1.9 years; 270 patients in the rosuvastatin developed diabetes, which was an risk increase of 26% compared to the placebo group (216 patients with new-onset diabetes; *P* = 0.01). In following meta-analyses from statin trials, it was shown that moderate-dose statin therapy conveyed a 11% increase in risk of diabetes compared to placebo,^47,48^ whereas high-dose statin therapy increased risk of diabetes by 23% compared to placebo.^48,49^ Furthermore, statins were associated with a modest increase in total body weight of 0.33 kg compared to placebo.^48^ These results were confirmed in genetic studies that showed that genetic inhibition of the *HMGCR* gene increased the lifetime risk of new-onset diabetes.^48,50^ This increase in diabetes risk is not limited to statins, since studies with the genetic proxies of ezetimibe (*NPC1L1*)^51^ and PCSK9 inhibitors (*PCSK9*),^50^ have shown consistent untoward results for new-onset diabetes. Another study has illustrated that approximately 1 mmol/L (1SD) genetically instrumented elevation in LDL-C was associated with a 21% (OR: 0.79; 95% CI: 0.71–0.88) lower risk of Type 2 diabetes.^52^ Interestingly, in the same study, 0.4 mmol/L (1SD) genetically instrumented elevation in HDL-C was associated with a 17% (OR: 0.83; 95% CI: 0.76–0.90) lower Type 2 diabetes risk.^52^

These results imply that agents increasing HDL-C may have a beneficial effect on diabetes risk. Major outcome trials with CETP inhibitors have confirmed this relationship. A preliminary report with the first year of follow-up from the Investigation of Lipid Level Management to Understand its Impact in Atherosclerotic Events (ILLUMINATE) trial showed reductions in glucose and insulin and a trend towards a reduction in new-onset diabetes.^53^ These findings led to an analysis from the four completed placebo-controlled CV outcome trials of CETP inhibitors (ILLUMINATE,^54^ Dal-OUTCOMES,^55,56^ ACCELERATE,^57^ and REVEAL^58^). In total, 73 479 participants from these trials were included in this meta-analysis.^59^ In the CETP inhibitor group, 960 patients developed new-onset diabetes compared with 1086 patients in the placebo group, a 12% reduction in diabetes risk (OR: 0.88, 95% CI: 0.81–0.96; *P* = 0.005). Furthermore, the Dal-OUTCOMES trial results showed that dalcetrapib also increased regression from diabetes at baseline to a non-diabetic state in follow-up by 25% (OR: 1.25; 95% CI: 1.06–1.49).^56^

As pointed out, higher HDL-C levels are associated with lower risk of diabetes, and so is CETP inhibition. Despite this previous evidence, the mechanism of the diabetes reducing effect of HDL-C is not fully unravelled yet. Cellular and rodent studies have shown that HDL particles, but also main HDL apolipoproteins, apoAI and apoAII, are known to increase insulin synthesis in and secretion from pancreatic beta islet cells.^60,61^ HDL enhances glucose uptake by skeletal muscle^62^ and prevents the skeletal muscle insulin resistance associated with cholesterol-induced activation of macrophages.^63^

In *in vitro* and rodent studies, HDL increased insulin synthesis as well as insulin secretion in the Ins-1E and MIN6 clonal beta-cell lines and in isolated mouse and rat pancreatic islets.^60,61^ HDL also inhibits apoptosis,^64^ reduces inflammation,^65^ increases insulin sensitivity,^62^ and a low level of HDL-C is associated with insulin resistance.^66^*In vivo*, ABCA1 loss-of-function mutation carriers show impaired insulin secretion.^67,68^ In contrast, a reduced Type 2 diabetes incidence was also found in heterozygous familial hypercholesterolaemia (FH) patients in the Netherlands.^69^ Individuals with heterozygous FH have only 50% of LDL receptors, therefore the LDL-C uptake by the pancreatic beta cell is reduced. Since free cholesterol accumulation in pancreatic beta cells reduces beta cell function,^70^ the reduced diabetes incidence in heterozygous FH patients provides a rationale for the association between up-regulation of the LDL receptor and diabetes risk. Conversely, CETP inhibition confers a decrease in pancreatic beta cell cholesterol content. Since CETP increases pre-beta HDL, which promotes cholesterol efflux via ABCAI/GI,^44,60^ this is an important additional hypothesis for the mechanism whereby CETP inhibition might reduce diabetes risk.

Thus, the increase in HDL following CETP inhibition may very well explain the reduction of diabetes risk as observed in the CVOT trials. Since diabetes is a strong risk factor for ASCVD, the effect of CETP inhibition on diabetes provides an additional mechanism by which CETP inhibitors can reduce ASCVD risk in the longer term.

## 6. Clinical development of CETP-lowering agents

Since the discovery of CETP as therapeutic target for ASCVD risk reduction, four CETP inhibitors have been tested in clinical outcome trials: torcetrapib, dalcetrapib, evacetrapib, and anacetrapib. The most recently developed CETP inhibitor obicetrapib has yet to be investigated in a large-scale Phase 3 programme, to be initiated towards the end of 2021.

### 6.1 Torcetrapib

Torcetrapib was the first CETP inhibitor developed after it was shown that CETP inhibition prevented development of atherosclerosis in CETP expressing rabbits.^22^ Subsequently, in early-phase clinical studies, torcetrapib showed increase of HDL-C by 60–100%, while concordantly lowering LDL-C by up to 20%.^54^ Its Phase 3 trial, ILLUMINATE, investigated the effect of torcetrapib 60 mg once daily in a randomized, double-blind fashion.^54^ In total, 15 067 patients were included randomized to either atorvastatin alone or torcetrapib plus atorvastatin. The primary outcome was defined as the time to first MACE, which was a composite of death from coronary heart disease (CHD), non-fatal MI, stroke, or hospitalization for unstable angina. The trial was terminated early because of increased incidence of death and CVD events in patients receiving torcetrapib. At the termination of the study on 2 December 2006, the median follow-up in each group was 550 days.

Compared with baseline levels, torcetrapib increased HDL-C by 72.1% (from 49 to 83 mg/dL; *P* < 0.001) and LDL-C was decreased by 24.9% (from 80 to 58 mg/dL; *P* < 0.001). Despite these favourable changes in lipid profile, there was an increased risk of CVD events [hazard ratio (HR): 1.25; 95% CI: 1.09–1.44; *P* = 0.001] and all-cause mortality (HR: 1.58; 95% CI: 1.14–2.19; *P* = 0.006).^54^ The HR estimates for the individual components of the composite outcome ranged from 1.35 for hospitalization for unstable angina (*P* = 0.001) to 1.08 for stroke (*P* = 0.74). In the torcetrapib group, compared with the atorvastatin-only group, there was an increased risk of death from both CVD causes (49 vs. 35) as well as other causes (40 vs. 20). For death from non-CVD causes, more patients in the torcetrapib group compared with the atorvastatin-only group died from infectious diseases (9 vs. 0) and cancer (24 vs. 14).

Remarkably, no single cause of CVD death explained the increased CVD risk. In the analysis of the negative outcome of the ILLUMINATE trial, multiple possible safety concerns surfaced. First, there was an increase of 5.4 mmHg in systolic blood pressure in the torcetrapib group. Second, at 12 months, 2.3% of patients receiving torcetrapib had potassium levels below 3.5 mmol/L, compared to only 0.6% in the atorvastatin-only group. Third, there were greater increases in sodium concentrations (1.39 mmol/L vs. 0.78 mmol/L) and bicarbonate (2.28 mmol/L vs. 1.93 mmol/L) in the torcetrapib group compared to the atorvastatin-only group. Post-hoc analyses showed an increased risk of death in patients treated with torcetrapib whose reduction in potassium or increase in bicarbonate was greater than the median change. Fourth, the QT interval was increased by a median of 3.3 ms at 12 months in the torcetrapib group and was decreased by 0.3 ms in the atorvastatin-only group (*P* < 0.001). Lastly, aldosterone 85th, 90th, and 95th percentiles were 8.6, 10.0, and 13.0 ng/dL, respectively, in the atorvastatin-only group and 9.5, 11.0, and 14.0 ng/dL, respectively, in the torcetrapib group (*P* < 0.001).

After careful assessment of all available data, it is most likely that the safety concerns observed with torcetrapib were a result of increases in aldosterone, cortisol, and endothelin-I, as well as profound changes in serum potassium and bicarbonate and, finally, significant increases in blood pressure. Notably, data from short-term Phase 2 studies already had indicated that torcetrapib raised both diastolic and systolic blood pressures.^71^ Results of following studies showed that torcetrapib increased the synthesis and secretion of both aldosterone and cortisol from adrenal cortical cells in tissue culture and increased expression of endothelin-1 in the artery wall.^72,73^ Furthermore, these findings were confirmed in animal models that lack CETP, confirming the off-target nature of these side effects.^74^

The observed off-target effects observed with torcetrapib resulted in careful assessment in of all other CETP inhibitors previously in development. All these agents underwent careful assessments in pre-clinical and clinical studies to exclude off-target toxicity. In these studies, none of the other inhibitor in the CETP inhibitor class showed notable off-target effects such as increase in blood pressure or increases in non-CVD mortality of morbidity.^11,75^ Even ambulatory blood pressure studies with dalcetrapib, anacetrapib, and evacetrapib showed no indication of clinically relevant effects on blood pressure or mineralocorticoid levels.^76,77^

### 6.2 Dalcetrapib

As described previously, evidence from observational studies into HDL-C and ASCVD events had shown an inverse relation between HDL-C plasma levels and ASCVD incidence. Therefore, dalcetrapib, which raised HDL-C levels by approximately 30% in multiple Phase 2 studies, without effect on LDL-C levels, blood pressure, or circulating neurohormones, was hypothesized to result in ASCVD risk reduction. The Phase 3 clinical outcome trial of dalcetrapib, Dal-OUTCOMES (Randomized, Double-blind, Placebo-controlled Study Assessing the Effect of RO4607381 on Cardiovascular Mortality and Morbidity in Clinically Stable Patients with a Recent Acute Coronary Syndrome), was set out to confirm this hypothesis.^55^

In the Dal-OUTCOMES trial, dalcetrapib was administered at a dose of 600 mg and compared to placebo in post-MI patients who were optimally treated according to guidelines. In total, 15 871 patients were included whom were followed for a median of 31 months. The primary endpoint consisted of MACE, which was a composite of death from CHD, non-fatal MI, ischaemic stroke, unstable angina, or cardiac arrest with resuscitation. At randomization, the mean HDL-C level was 1.1 mmol/L (42 mg/dL), and the mean LDL-C level was 2.0 mmol/L (76 mg/dL). During the trial, HDL-C levels increased from baseline by 4–11% in the placebo group and by 31–40% in the dalcetrapib group [mean ∼1.8 mmol/L (68 mg/dL) at Month 36]. As expected, dalcetrapib had a negligible effect on LDL-C and apoB levels. The trial was terminated for futility by recommendation of the independent data and safety committee after a pre-specified interim analysis that included 1135 primary endpoint events (71% of the projected total number). Dalcetrapib did not show any effect on the primary endpoint compared to placebo [HR: 1.04; 95% confidence interval (CI): 0.93–1.16; *P* = 0.52] and did not affect any component of the primary endpoint or all-cause mortality.^55^ In addition, there was no association between the observed change in HDL-C and the risk of the primary endpoint.

Overall, dalcetrapib had no appreciable side effects and was well tolerated. The mean systolic blood pressure was slightly higher with dalcetrapib compared with placebo (0.6 mmHg; *P* < 0.001). There were no significant between-group differences in diastolic blood pressure, pulse rate, and plasma levels of aldosterone, potassium, or bicarbonate. In summary, no association was shown between increased HDL-C and reduced CV risk among the patients evaluated in Dal-OUTCOMES; however, there were no safety issues seen with dalcetrapib.

### 6.3 Evacetrapib

Evacetrapib, in contrast to dalcetrapib, did lower LDL-C and apoB levels next to increasing HDL-C in its early-phase clinical trials.^74^ The Phase 3 trial, ACCELERATE (The Assessment of Clinical Effects of Cholesteryl Ester Transfer Protein Inhibition with Evacetrapib in Patients at a High-Risk for Vascular Outcomes), investigated evacetrapib in 12 092 secondary prevention patients.^57^ Patients had to be treated with a statin and were required to have an HDL-C level below 2.1 mmol/L (80 mg/dL) to be eligible for study enrolment. In addition, patients had to have an LDL-C level at enrolment that was no more than 10 mg/dL (0.25 mmol/L) above their treatment target of 1.8 mmol/L (70 mg/dL) or 2.6 mmol/L (100 mg/dL). In addition to their baseline therapy, patients were randomized to either 130 mg evacetrapib or placebo. The primary endpoint consisted of MACE, which was a composite of death from CV causes, MI, stroke, coronary revascularization, or hospitalization for unstable angina.

The data and safety monitoring board recommended the trial to be terminated due to futility after a median of 26 months of treatment, which was after 1363 of the planned 1670 primary endpoints had been reached. In the evacetrapib group, a primary endpoint event occurred in 12.9% of patients compared to 12.8% of patients in the placebo group (HR: 1.01; *P* = 0.91). There was no significant difference between secondary endpoints. The incidence of all-cause mortality (unadjusted for multiple comparisons) was significantly lower with evacetrapib than with placebo (*P* = 0.04).

The fact that LDL-C-lowering therapies such as evacetrapib reduce ASCVD risk proportional to the achieved LDL-C reduction, but also to the duration of therapy, explains the lack of significant results for MACE reduction in the ACCELERATE trial.^78^ The median follow-up period was no longer than 26 months. Although there was a supposedly 31% decrease in mean LDL-C levels to 1.41 mmol/L at 3 months in the evacetrapib group, these LDL-C levels were measured with a direct assay, whereas apoB reductions were considerably less pronounced (16%), suggesting that the direct assay has overestimated the LDL-C reduction. Considering the median follow-up and the observed apoB reduction in the ACCELERATE, the expected major adverse CVD event reduction was within the 95% CI for the effect size that was reported in the trial (HR: 0.97; 95% CI: 0.85–1.10) for the primary MACE endpoint.^34^ In comparison, in the IMPROVE-IT trial testing ezetimibe, which was performed in a similar post-acute coronary syndrome population as ACCELERATE, apoB reductions were in the same range (11.3 mg/dL for ezetimibe vs. 12.1 mg/dL for evacetrapib) and the ezetimibe-treated patients did not achieve a separation of the Kaplan–Meier curves until at least 3 years of treatment duration. Thus, it is likely that the ACCELERATE study was too short to detect a significant reduction in MACE.^6^ In fact, anacetrapib also required 3 years to achieve a separation of the Kaplan–Meier curves in the REVEAL trial. While evacetrapib did not demonstrate overall major adverse CVD events (MACE) reduction at 26 months, there were no significant safety signals, and a nominally significant (*P* = 0.04) reduction in total mortality was observed.

### 6.4 Anacetrapib

The most recent CETP inhibitor tested in clinical trials was anacetrapib. The primary results from its Phase 3 trial REVEAL, which was the largest Phase 3 trial for CETP inhibition to date, were published in 2017.^58^ REVEAL was a randomized, double-blind, placebo-controlled trial in 30 449 participants with ASCVD with a mean of 4.1 years follow-up, which compared anacetrapib 100 mg once daily to placebo. Adults were eligible when the total cholesterol concentration at randomization was below or equal to 4 mmol/L (155 mg/dL). Consequently, at baseline, the mean LDL-C level was a surprisingly low 1.6 mmol/L (61 mg/dL) and patients had a mean HDL-C level of 1.0 mmol/L (40 mg/dL). Patients had a mean age of 67 years, 88% of patients had a history of CHD, 22% had a history of cerebrovascular disease, 8% had peripheral artery disease, and 37% had diabetes. The primary endpoint was defined as the first major coronary event, which was a composite of coronary death, MI, or coronary revascularization. Secondary outcomes were major atherosclerotic events (a composite of coronary death, MI, or ischaemic stroke), ischaemic stroke, and major vascular events (a composite of major coronary events or ischaemic stroke).

In REVEAL, during a median follow-up period of 4.1 years, the primary endpoint occurred in significantly fewer patients in the anacetrapib group than in the placebo group (10.8% vs. 11.8% of patients; rate ratio 0.91; 95% CI: 0.85–0.97; *P* = 0.004).^58^ In addition, there was a significant reduction of major coronary events that occurred more than 1 year after randomization in the anacetrapib group (rate ratio: 0.88; 95% CI: 0.81–0.95; *P* = 0.001). At the trial midpoint at 2 years, mean LDL-C, which was measured by a direct assay, was 1.0 mmol/L (38 mg/dL), 0.7 mmol/L lower compared to the mean LDL-C of 1.7 mmol (64 mg/dL) in the control group (relative difference 41%). LDL-C was also analysed by the golden standard, beta quantification, in a subgroup of 2000 patients, which revealed that the actual LDL-C reduction was remarkably less. Beta quantification showed an absolute LDL-C difference of only 0.3 mmol/L (11 mg/dL; relative difference 17%) at trial midpoint between anacetrapib and placebo. In earlier studies, it was shown that the Friedewald formula and the direct assays underestimate actual LDL-C levels, resulting in an overestimation of the percentage change from baseline in LDL-C levels measured by these methods.^79,80^ This problem especially occurs when LDL-C levels are very low. Confirmatory, the mean apoB was 0.12 g/L (12 mg/dL) lower in the anacetrapib group compared to placebo, which is a relative difference of 18%, compatible with the 17% reduction of LDL-C using the beta quantification method.

Overall, there were no significant safety signals. Compared to placebo, treatment with anacetrapib did not result in significant differences in rates of death from CVD causes, death from all non-CVD causes, or all-cause mortality. Also, there were no significant between-group differences in the incidence of cancer or of other serious adverse events. As observed with the other CETP inhibitors, new-onset diabetes mellitus occurred less frequently in the anacetrapib group compared to the placebo group (5.3% vs. 6.0%; rate ratio: 0.89; 95% CI: 0.79–1.00; *P* = 0.0496). In contrast to reports from some Mendelian randomization studies, there was no evidence of adverse effects associated with anacetrapib on macular degeneration.

**Figure 4:**
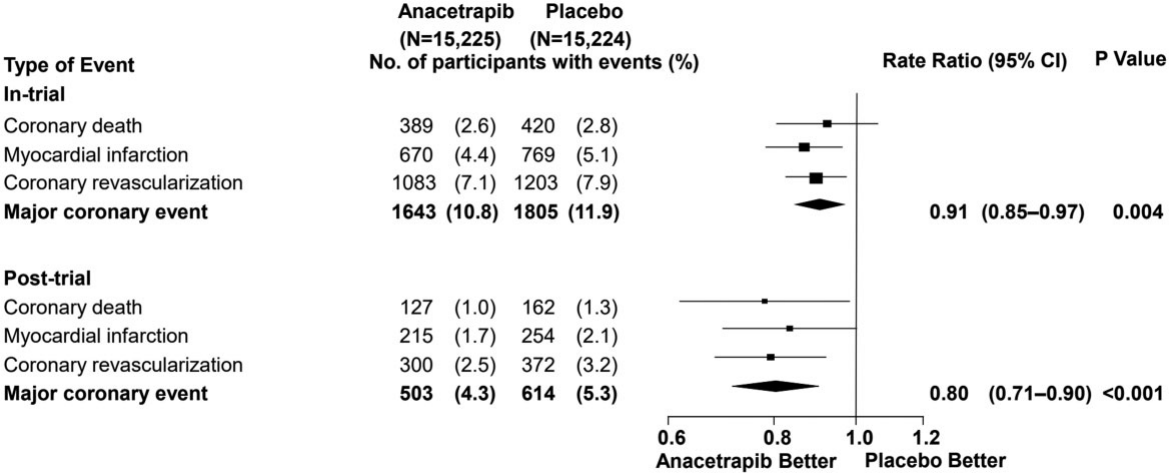
REVEAL—components of major coronary event during 6.4 years of follow-up. Depicted is the primary outcome and its components of the in-trial and post-trial follow-up of the REVEAL study. Adapted from REVEAL Collaborative Group, MDP477. The Effects of Anacetrapib Therapy on Occlusive Vascular Events During Post-Trial Follow-Up of the REVEAL Randomized Trial, 2019 American Heart Association Scientific Sessions. Reprinted with permission from REVEAL, Randomized EValuation of the Effects of Anacetrapib through Lipid-modification.

**Figure 5:**
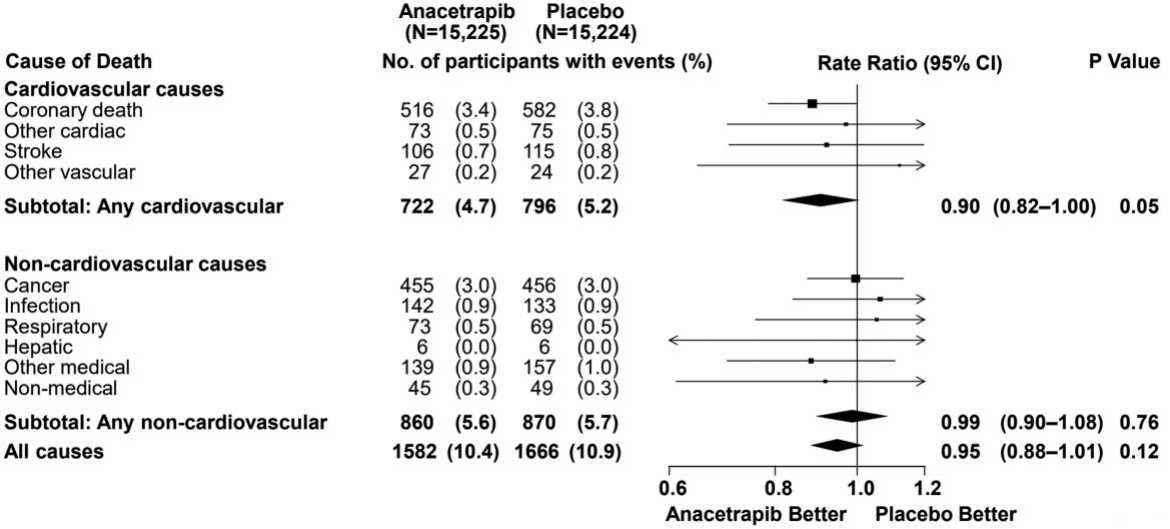
REVEAL—effect on mortality during 6.4 years of follow-up. Depicted are the secondary outcomes and their components of the post-trial follow-up of the REVEAL study. Adapted from REVEAL Collaborative Group, MDP477. The Effects of Anacetrapib Therapy on Occlusive Vascular Events During Post-Trial Follow-Up of the REVEAL Randomized Trial, 2019 American Heart Association Scientific Sessions. Reprinted with permission from REVEAL, Randomized EValuation of the Effects of Anacetrapib through Lipid-modification.

Since anacetrapib has been shown to have a very long terminal half-life time in earlier preclinical and early-phase clinical studies due to adipose tissue accumulation, patients were followed up after the end of the treatment period in REVEAL. Follow-up for clinical outcomes was continued for a median of 2.3 years by telephone interviews and medical record review to investigate longer-term safety and efficacy. This allowed the randomization in REVEAL to be maintained during the entire post-trial follow-up period. An additional analysis of the primary composite endpoint, major coronary events, was performed after the post-trial follow-up and showed a significant 20% reduction of these events compared to the initial trial endpoint (*Figure 4*; *P* < 0001). In addition, after the post-trial follow-up, all individual components of the composite endpoint occurred significantly less in the anacetrapib group compared to the placebo group (*Figure 4*). There was also a significant and clinically relevant effect on the rates of CV death in favour of anacetrapib (*Figure 5*).

In summary, the absolute reduction in major coronary events seen in the clinical trial after 4 years doubled during the post-trial follow-up of more than 2 years. The initial modest net benefit in major coronary events seen with anacetrapib in REVEAL could be explained by the already very low LDL-C (and apoB) levels at baseline, i.e. 1.6 mmol/L, and therefore, a modest absolute LDL-C differential. Nonetheless, no clinically relevant adverse effects were observed and no safety issues occurred during the trial. During the post-trial follow-up, between-group differences in risk of CVD death emerged, which were not observed in the trial period. These results are consistent with the observed non-HDL-C and apoB reduction with anacetrapib, and in line with the proportional risk reduction expected per amount of non-HDL-C and apoB lowering.^34,58^

### 6.5 Obicetrapib

Obicetrapib (also known as TA-8995) is the most recent addition to the CETP inhibitor class. In the phase 1 trials in healthy subjects, obicetrapib showed already near maximal CETP inhibition with daily doses of only 2.5 mg.^43^ The Phase 2 dose finding trial of obicetrapib, TULIP (TA-8995-03: Its Use in Patients with Mild Dyslipidaemia) was performed in 364 participants with dyslipidaemia receiving obicetrapib up to 10 mg once daily for 12 weeks.^44^ The 5 mg dose achieved reductions in LDL-C (measured with the gold standard beta quantification) and apoB of 45% and 34%, respectively, whereas HDL-C was increased with 157%. In the single ascending and multiple ascending dose studies with obicetrapib, which investigated doses up to 150 mg and 25 mg, respectively, no clinically significant effects were observed on vital signs, including blood pressure or on aldosterone, sodium, bicarbonate, high-sensitivity C-reactive protein or endothelin-I levels. No other serious adverse or toxic effects were reported in the early phase trials.

## 7. Clinical need for additional LDL-C-lowering therapies

The global burden of ASCVD is high and rising. According to the World Health Organization Global Health Estimates, ischaemic heart disease and stroke are the leading and second cause of death, respectively.^81^ In 2019, ∼15.1 million deaths were attributed to ischaemic heart disease and stroke across the globe, which amounted to an increase of 24% compared to 2000.^81^ In light of the rise in ASCVD risk factors, such as diabetes mellitus and obesity, the global burden of ASCVD is expected to increase further. In addition, due to socio-economic changes, the burden of ASCVD is rising even more in low- and middle-income countries than in high-income countries.^82^

The prevention of ASCVD consists of promoting healthy lifestyle and reducing individual ASCVD risk factors such as LDL-C/apoB, hypertension, and diabetes.^83,84^ For LDL-C reduction, statins and ezetimibe are most frequently used for across all risk groups. In higher risk patients, PCSK9 inhibiting monoclonal antibodies can be used on top of statins and ezetimibe. Unfortunately, statin uptake is hampered by statin intolerance,^4^ whereas PCSK9 inhibitors are frequently discontinued, and due to their high price not widely available. In the most recent ESC/EAS guidelines as well as in the AHA/ACC guidelines from 2018, LDL-C treatment targets for the different ASCVD risk groups are clearly defined.^83,84^ As highlighted previously, despite current treatment guidelines, a recently conducted EU-wide cross-sectional observational study revealed that overall risk-based 2019 LDL-C target attainment was observed in in just 18% of very high-risk patients.^3^ In the different ASCVD risk groups low, moderate, high, and very high risk, the LDL-C goal attainment was 63%, 75%, 63%, and 39%, respectively. Among very high-risk patients receiving statin monotherapy, goal attainment was 14%, 16%, and 22% in those receiving low-, moderate-, and high-intensity statins, respectively.

The prevalent gap between guideline-recommended LDL-C targets and target attainment emphasizes the need for additional non-statin LDL-C-lowering therapies, especially for patients at the highest risk.^3^ As shown in statin, ezetimibe as well as PCSK9 trials, but also in the REVEAL study, the benefit from treatment with LDL-C-lowering therapies is proportional to the absolute magnitude of LDL-C/apoB reduction and the duration of the LDL-C-lowering therapy.^1,58,85^ For this benefit, it should be irrelevant whether LDL-C lowering is achieved by statins, ezetimibe, PSCK9 inhibition, or CETP inhibition. CETP inhibition could be an interesting therapeutic option as add-on to statin-therapy or in partially or completely statin intolerant patients. Importantly, the new generation CETP inhibitors have a favourable safety profile. While anacetrapib was already proven effective in reducing ASCVD in REVEAL, obicetrapib has been shown to achieve twice the LDL-C and apoB reduction compared to anacetrapib, and thus potentially a greater reduction in ASCVD risk.

## 8. Conclusions

CETP inhibition is effective in reducing LDL-C, apoB, and increasing HDL-C. CETP inhibition is often cited as an example in which LDL-C/apoB reduction does not uniformly result in a reduction in major adverse cardiovascular events. However, recent evidence through Mendelian randomization studies and more importantly clinical trial data with anacetrapib have confirmed that this class of agents is demonstrating effective LDL-C reductions in combination with statins. In addition, CETP inhibitors lower the risk of new-onset diabetes and improve glucose tolerance as well as insulin sensitivity. Despite off-target effects of the first CETP inhibitor torcetrapib, other CETP inhibitors were safe. Obicetrapib has been shown to provide significant LDL-C and apoB reductions, and if successfully tested in its Phase 3 programme, could become the first clinically available CETP inhibitor.

**Take home message box**
